# Supplementary material for: Inhibition of NHEJ repair by type II-A CRISPR-Cas systems in bacteria
Source: Nat Commun. 2017 Dec 12;8:2094. doi: 10.1038/s41467-017-02350-1 (PMC5727150; doi:10.1038/s41467-017-02350-1)
Supplement: Supplementary file 1 — Supplementary Information [file 41467_2017_2350_MOESM1_ESM.pdf]

Supplementary Figures

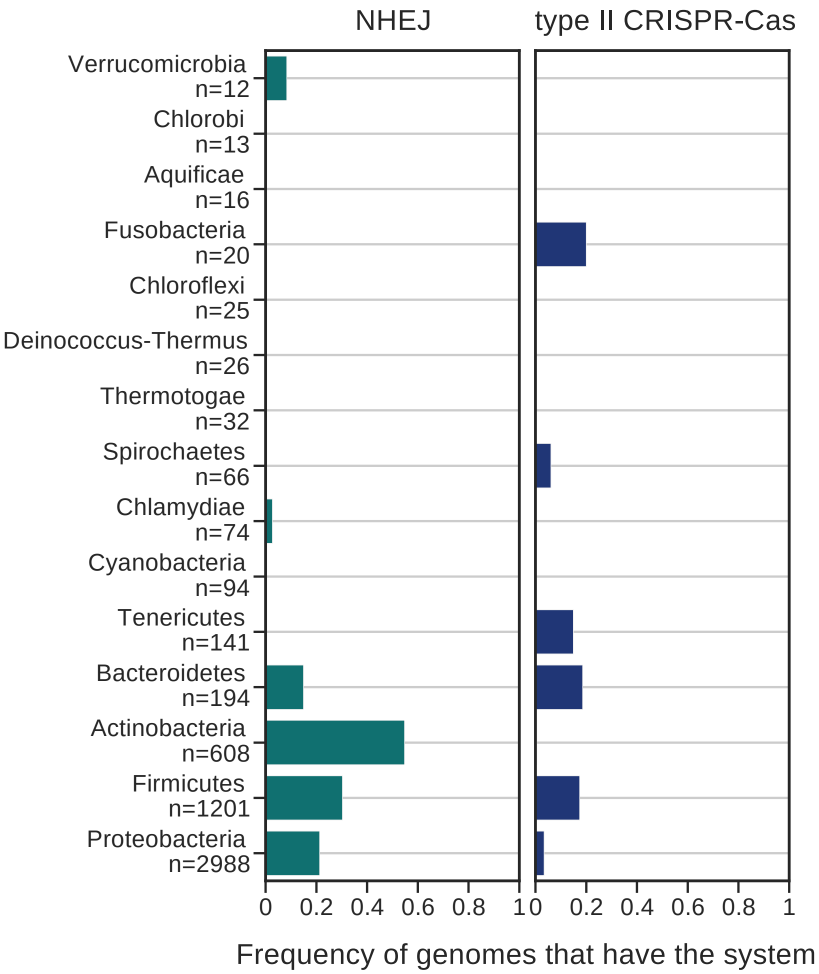

Supplementary Figure 1: Frequency of NHEJ and CRISPR-Cas systems by clades.

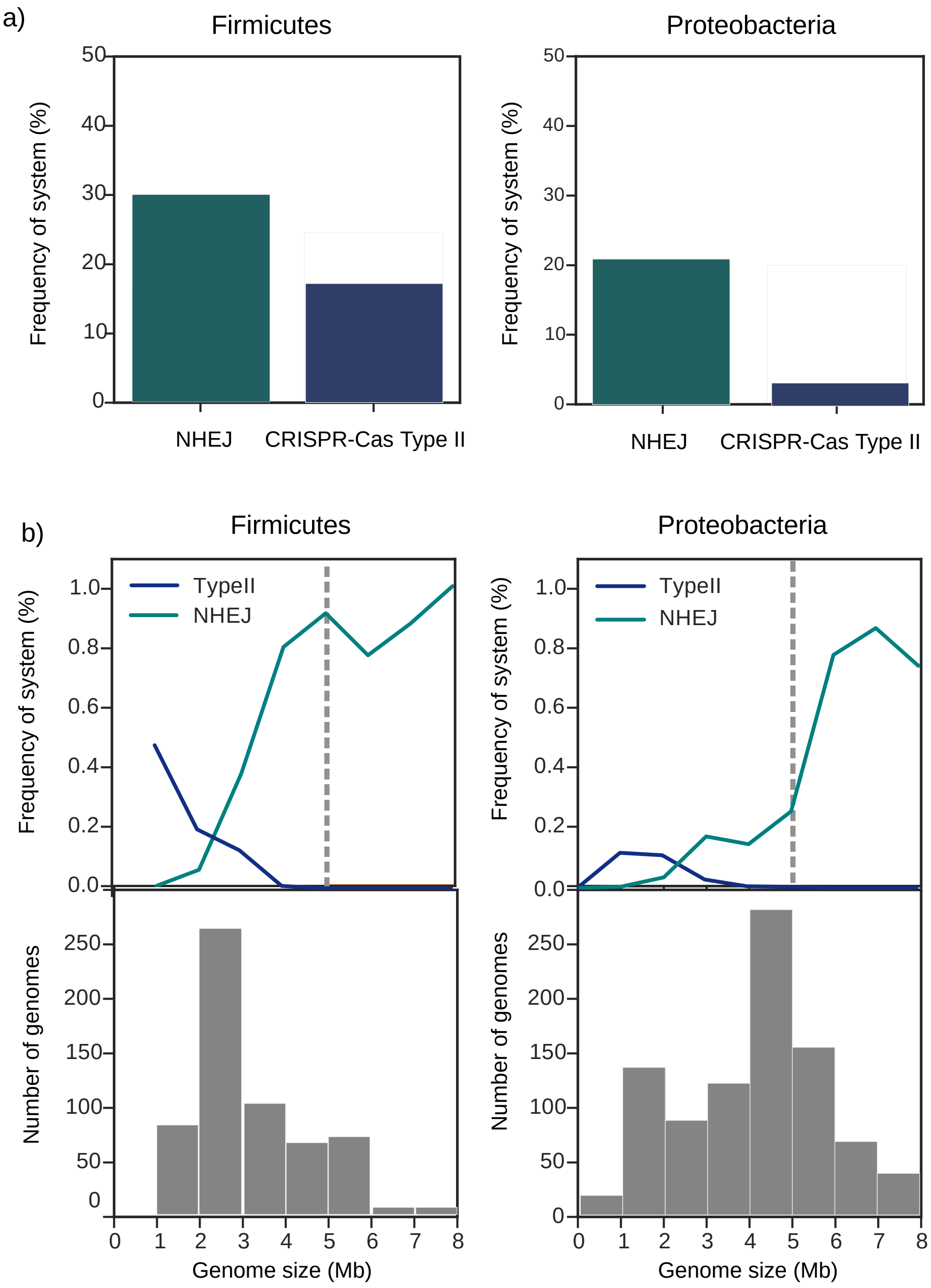

Supplementary Figure 2: Distribution of NHEJ and CRISPR-Cas systems in the genomes of Firmicutes and Proteobacteria. a, Frequency of NHEJ and CRISPR-Cas systems in the 1201 Firmicutes and 2988 Proteobacteria genomes. **b,** Frequency of NHEJ and CRISPR-Cas systems in function of genome size. The histogram on the bottom represents the distribution of genome sizes in each clade. The frequency represents the frequency of genomes carrying a system within the genome size range. Vertical line corresponds to the size cut-off (5Mb).

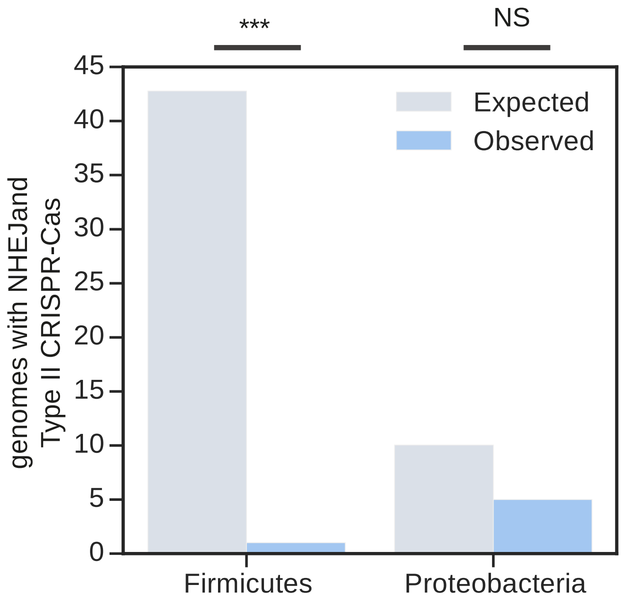

Supplementary Figure 3: Associations between NHEJ and Type II CRISPR-Cas systems in Firmicutes and Proteobacteria genomes. The expected values are the product of the marginal row and column totals divided by the grand total of the contingency tables. Statistics were calculated using Fisher's Exact Test. ** P< 0.05, *** P< 0.01

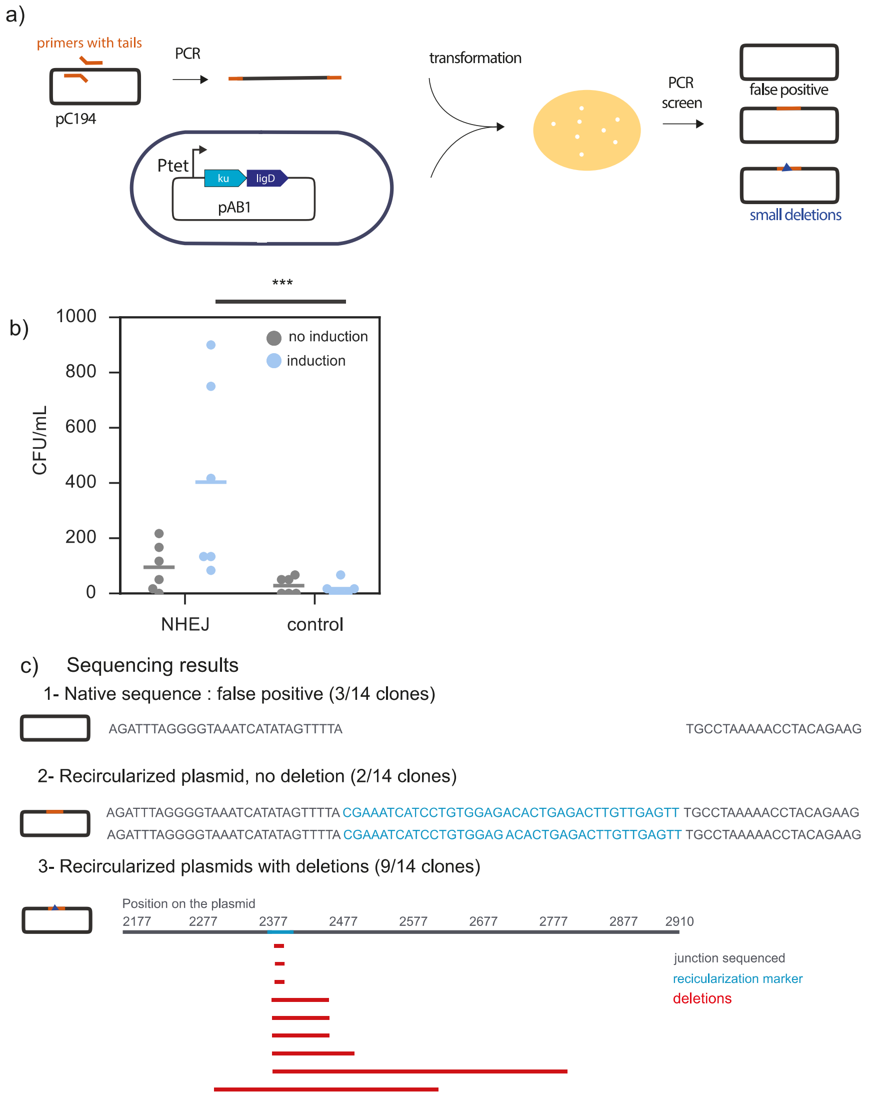

Supplementary Figure 4: NHEJ system of *B. subtilis* is functional in *S. aureus*. a, Introduction of a linearized plasmid providing resistance to chloramphenicol (pC194) into cells carrying the NHEJ system (pAB1) either in the presence or absence of aTc (induction) in *S. aureus*. Only cells that re-circularized the plasmid could form colonies on chloramphenicol plates. **b,** The CFU of five replicates is represented, error bars corresponds to s.e.m. *** P< 0.01 Wilcoxon test **c,** Sequencing results of transformants recovered after NHEJ induction. The primers used to linearize pC194 were designed to carry 5' tails (in blue). If plasmids were recircularized by the NHEJ system, we expected to find those tails sequence at the junction. Results are shown for clones recovered after NHEJ induction. Blue corresponds to overhangs and red to deletions. The insertion of the intact tail sequences at the junction was observed in 2/14 colonies recovered after NHEJ induction. In 9/14 colonies, small deletions were observed. The remaining 3/14 colonies were false positives. In contrast, all colonies obtained in the control experiment corresponded to the pC194 plasmid that was likely present in trace amounts in the PCR products used for the transformation.

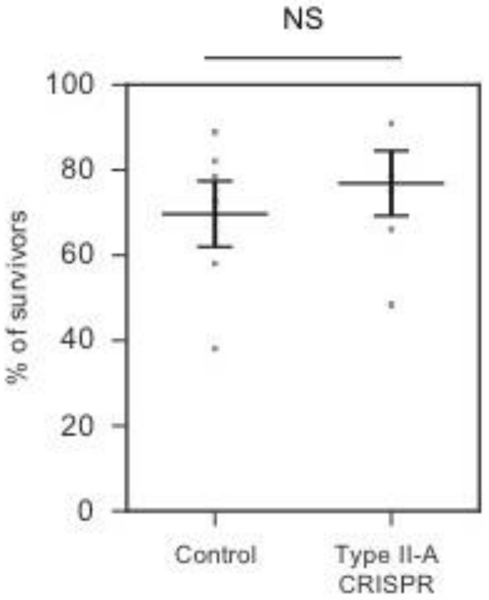

Supplementary Figure 5 : Irradiation of *S. aureus* cells

S. aureus cells carrying a type II-A CRISPR-Cas systems or a control plasmid were irradiated. The survival rate was determined as the ration of CFUs obtained for irradiated over non-irradiated cells. No significant difference was observed (n=5 Wilcoxon, P=0.5).

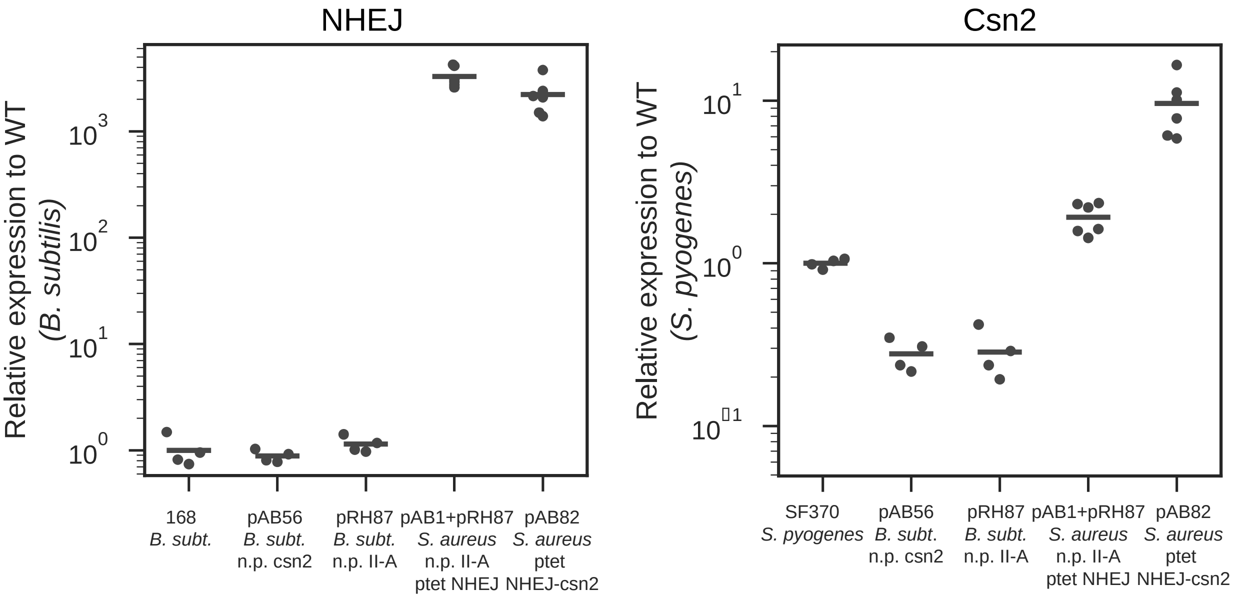

Supplementary Figure 6 : Expression level of NHEJ and Csn2

Expression of NHEJ and Csn2 was measure using q-PCR in strains used in the study (see Supplementary Table 3 for a description of the plasmids). Expression was normalized to the 16s_rRNA expression in each strain measured using the same set of primers. Expression is shown relative to the wild-type: *B. subtilis* 168 for NHEJ and *S. pyogenes* SF370 for Csn2.

Supplementary Tables

Supplementary Table 1: Detected systems and their frequencies by genomes

Systems	Bacterial Genomes (2482)	Frequency (%) (Bacteria)
Type I CRISPR-Cas	1584	28.5
Type II CRISPR-Cas	384	6.9
Type III CRISPR-Cas	306	5.5
NHEJ	1376	24.7

Supplementary Table 2: Plasmids used in this study

**Plasmids from other studies**		**Described in**
pC194/pE194	Replicative plasmid in *S. aureus*, respectively CmR, ErmR,	[1,2]
pT181	Replicative plasmid in *S. aureus*, tetR	[3]
pMUTIN4	Integrative plasmid in *B. subtilis*, ErmR	[4]
pNZ123	Replicative plasmid in *S. thermophilus*, CmR	[5]
pLZ12	Replicative plasmid in *S. aureus* and *E. coli*	[6]
pCN57	Plasmid with a working GFP in *S. aureus*	[7]
pRH87	pC194 with type II-A CRISPR-Cas system from *S. pyogenes* with one spacer in the CRISPR array	[8]
pRH61,pRH62,pRH63	same as pRH87 but without respectively *cas1*,*cas2*, *csn2*	[8]
pRH121	same as pRH87 but with *dcas9* instead of *cas9*	[8]
pDB114	pC194 with *cas9* and reprogrammable spacer	[9]
pWJ153	pE194 Ptet inducible target vector	[9]

Plasmids created for this study		Insert (Primer ; Template)		Vector (Primer ; Template)		Cloned in
pAB1	pE194 with NHEJ system from *B. subtilis* under tet promoter (Ptet)	AB+AB4	*B. subtilis* genomic DNA	AB1+AB2	pWJ153	*S. aureus*
pAB2	pT181 with a protospacer from PhiNM4 phage			AB9+AB10	pT181	*S. aureus*
pAB12	pMUTIN with ykoV homologies (to build ykoV mutant in *B. subtilis*)	AB69+AB70	*B. subtilis* genomic DNA	AB67+AB68	pMUTIN 4	*E. coli*
pAB13	pMUTIN with ykoU homologies (to build ykoU mutant in *B. subtilis*)	AB73+AB74	*B. subtilis* genomic DNA	AB71+AB72	pMUTIN 4	*E. coli*
pAB17	pE194 with ligD under tet promoter (Ptet)			AB85+AB86	pAB1	*S. aureus*
pAB18	pE194 with Ku under tet promoter (Ptet)			AB87+AB88	pAB1	*S. aureus*
pAB23	pLZ12 with NHEJ under tet promoter (Ptet)	AB95+AB96	pAB1	AB97+AB98	pLZ12	*E. coli*
pAB24	pLZ12 with Ku under tet promoter (Ptet)	AB95+AB96	pAB18	AB97+AB98	pLZ12	*E. coli*
pAB25	pLZ12 with ligD under tet promoter (Ptet)	AB95+AB96	pAB17	AB97+AB98	pLZ12	*E. coli*
pAB62	pLZ12 with GFP under tet promoter (Ptet)	ACV69+ACV70	pCN57	ACV64+ACV65 ACV67+ACV68	pAB23	*E. coli*
pAB66	pNZ123 with NHEJ under P8 constitutive *S. thermophilus* promoter	AB244+AB245 AB246+AB247	pAB1 ; *S. thermophilus* DNA	AB242+AB243	pNZ123	*E. coli*
pAB69	pNZ123 with GFP under P8 constitutive *S. thermophilus* promoter	AB50+AB251 AB252+AB253	pCN57 ; *S. thermophilus* DNA	AB248+AB249	pNZ123	*E. coli*
pAB56	pC194 with csn2 under type II-A natural promoter	Reciculrized		AB224+AB225	pRH87	*S. aureus*
pAB81	pAB1 with csn2 cloned directly after ligD	AB280+AB281	pAB56	AB278+AB279	pAB1	*S. aureus*
pAB82	pAB1 with csn2 cloned with the promoter of the cas operon of *S. pyogenes*	AB284+AB285	pAB56	AB282+AB283	pAB1	*S. aureus*
pMD021	pC194 with *cas9* and spacer targeting PhiNM4	D035+D036	Hybridized primers	Digested with Bsa1	pDB114	*S. aureus*

Supplementary Table 3: Primers used in the study

AB1	GCTCTTTCCGTGGTTCATATTTATCAGAGCTCGTGCTATAAT
AB2	GAGTACGATTCATTTGATATGCCTCCTCTAGGTCATTTGATATGCCTCCG
AB3	CAAATGACCTAGAGGAGGCATATCAAATGAATCGTACTCCTTCTCTTCAC
AB4	GATAAATATGAACCACGGAAAGAGCTGACTTCATTAG
AB9	AAAAATGTTTTAACACCTATTAACGTAGTATGGAGTGGCTAGCATTTTGC
AB10	CCATACTACGTTAATAGGTGTTAAAACATTTTTAAACTGCTTTTCAGAAC
AB67	AGTCCCAGGTGTACCTGATCAGAAAACCGCCTCGCG
AB68	ACAAAGTCAACAATCTCAACGATTCTCCGTGGGAACAAACGG
AB69	GTTTGTTCCCACGGAGAATCGTTGAGATTGTTGACTTTGTTCAGCTTCAG
AB70	TCACCGCGAGGCGGTTTTCTGATCAGGTACACCTGGGACTTGAG
AB71	ACTATGCGCCGGATTTTGTTAGAAAACCGCCTCGC
AB72	TGTTAAGTATTGAACAGCTGGATTCTCCGTGGGAACAAACG
AB73	GTTTGTTCCCACGGAGAATCCAGCTGTTCAATACTTAACAATTCTCCAAG
AB74	TCACCGCGAGGCGGTTTTCTAACAAAATCCGGCGCATAGTCC
AB85	CGCATGGCGTTTACCATGCA
AB86	GGTCATTTGATATGCCTCCGG
AB87	GAAGTCAGCTCTTTCCGTGGTTC
AB88	GCGCTATGATGTGCCGGAG
AB95	CTAATGAATTCATCTGCAGGAAAGAAATTAGATAAATCTCTCATATCTTTTATTCAATAATCGCAT
AB96	GGTCGTCAGACTGATGGGCCAATTATAGCACGAGCTCTGATAAATATGAACCAC
AB97	TCAGAGCTCGTGCTATAATTGGCCCATCAGTCTGACGAC
AB98	GAGATTTATCTAATTTCTTTCCTGCAGATGAATTCATTAGGATCCAGA
AB224	ATTTACATGGTGAAAGAAATAATTGTATTGCAAACTCC
AB225	TTGCCTCCTAAAATAAAAAGTTTAAATTAAATCC
AB242	ACTTCGAACTAGCAATACTGCTCTCTAGAGAATTCAGTACTGGATCT
AB243	TTCCGTGGTTCATATTTATCCTCAAGCTTCTCGAGTGCATATTTTCG
AB244	CTCGTGCGAGGTTTTTACATATGAATCGTACTCCTTCTCTTCACACTAAAG
AB245	ATGCACTCGAGAAGCTTGAGGATAAATATGAACCACGGAAAGAGCTGAC
AB246	GTACTGAATTCTCTAGAGAGCAGTATTGCTAGTTCGAAGTCATCCTTTTTTATAGG
AB247	AGAGAAGGAGTACGATTCATATGTAAAAACCTCGCACGAGTAGTTATTT
AB248	CGAACTAGCAATACTGTAAGAGCTCTCTAGAGAATTCAGTACTGGATCT
AB249	CAAATAAGGCGCGCCTATTCCAAGCTTCTCGAGTGCATATTTTCG
AB250	CTCGTGCGAGGTTTTTACATATGAGTAAAGGAGAAGAACTTTTCACTGGAG
AB251	ATATGCACTCGAGAAGCTTGGAATAGGCGCGCCTTATTTGTATAGTT
AB252	ACTGAATTCTCTAGAGAGCTCTTACAGTATTGCTAGTTCGAAGTCATCCT
AB253	AGTTCTTCTCCTTTACTCATATGTAAAAACCTCGCACGAGTAGTTATTT
AB278	GGTATAATACTCTTAATAAAAAGTCAGCTCTTTCCGTGGT
AB279	GTAAATTTGCCTCCTAAAATCATTAGTCAGCTCTTTTTCTTCAACTGATG

AB280	AGAAAAAGAGCTGACTAATGATTTTAGGAGGCAAATTTACATGGTGAAAGAA
AB281	ACCACGGAAAGAGCTGACTTTTTATTAAGAGTATTATACCATATTTTTAGTTATTAAG
AB282	AAATGCAGTAATACAGGGGCTCGTGCTATAATTATACTAATTTTATAAGGAGG
AB283	CAAAAAATATTACCCAATACGCTCTGATAAATATGAACCACGGAAAGAG
AB284	TGGTTCATATTTATCAGAGCGTATTGGGTAATATTTTTTGAAGAGATATTTTGAAAAAG
AB285	TTAGTATAATTATAGCACGAGCCCCTGTATTACTGCATTTATTAAGAG
ACV64	ACACATGGCATGGATGAACTATACAAATAATTTCTAAATAAGAATATTTGGAGAGCACCGTTC
ACV65	AGCATAACCTTTTTCCGTGATGGTA
ACV66	TTGATATGCCTCCTCTAGGTCATTTG
ACV67	TATAAATTTAACGATCACTCGTTACCATCACGG
ACV68	TCCAGTGAAAAGTTCTTCTCCTTTACTCATTTGATATGCCTCCTCTAGGTCATTTG
ACV69	ATGAGTAAAGGAGAAGAACTTTTCACTGGA
ACV70	TTATTTGTATAGTTCATCCATGCCATGTGTAAT
D035	AAAAATGTTTTAACACCTATTAACGTAGTATGTTTTAGAGCTATGCTGTTTTGA
D036	ATACTACGTTAATAGGTGTTAAAACATTTTTGTTTTGGGACCATTCAAAACAGC
B329	ACACTGAGACTTGTTGAGTTTGCCTAAAAACCTACAGAAG
B330	CTCCACAGGATGATTTCGTAAAACTATATGATTTACCCCTAAATCT
A9	TCAACGCACAATAAATTTTCTCGGC
A10	TACTTAAAAGAAATTGATCCAACCG
AB103	GCCCTCGAGTTGACAAGGACAGTTATTG
AB104	CAATTCGAATCTTGATTTGCTGTC

Supplementary Table 4: Example of spacers acquired during adaptation experiments of *S. aureus* against the phage PhiNM4

AGTTAAAAAGAATTTAAAGTCAAGAAGTA
ATGTTGATGGATCGTATCAAAGCGACATAC
AGGAATTGAGACACCTCAATATATACTTGC
ACACAAAGAAGTACATCAAGGGACAATTAC
CGAGCAAAGTTTCATCCGTTTAAATCAATA
TTAACGGTATGGAAGAAGCGAGTATCAATA
TACCGAATGAATTTTTAAAATATTCAGGCA
TCTTAAAGTTATTGAAGAAAGGTTATAACA
GGCAATGTTATTTTATCGGATTTTAAAAAC
GCTAATGACAGACCATTATTTGATGCTAAC
GACAAAATCGAACTATCATTAAAAGTTAAA
GCTATAGACGGAAGTTTCAACTTATTATAA
ACGACAGATATACGTCAGCGATTTATAATC
ACCGAATGAATTTTTAAAACATTCAGGCA
CAAATCTATTCAAGATACTATCGAAGCTGT

Supplementary Table 5: Primers used for RT-qPCR

Targeted genes	Primer name	Sequences (5’ to 3’)
Ku	LC1340_Ku_For	GGATCGATCAGCTTCGGATTAG
Ku	LC1341_Ku_Rev	TGGTGCGTGATCCTCTTTATG
Csn2	LC1342_csn2_For	GCAAACTCCGATGAAAGACTTG
Csn2	LC1343_csn2_Rev	ACCGCCTCTTAATGGAATCG
16s_rRNA	LC1344_16s_For	AGGCAGCAGTAGGGAATCTT
16s_rRNA	LC1345_16s_Rev	GCTGCTGGCACGTAGTTAG

Supplementary Notes

Supplementary Note 1: Functionality of NHEJ system from *B. subtilis in S. aureus*

NHEJ system from *B. subtilis* is functional in *S. aureus*

We first tested the functionality of the NHEJ system from *B. subtilis* in *S. aureus*. To do so, we cloned *ku* and *ligD* from *B. subtilis* into the staphylococcal vector pE194 under a tetracycline-inducible promoter Ptet (plasmid pAB1) and introduced it in RN4220 *S. aureus* cells. We then electroporated a linearized plasmid providing resistance to chloramphenicol (plasmid pC194) either in the presence or absence of anhydrotetracycline (aTc). Only cells that re-circularized the plasmid can form colonies on chloramphenicol plates (Supplementary Figure 4.a). We obtained 5 times more colonies when the NHEJ system was induced compared to the uninduced control (Supplementary Figure 4.b). Colonies were checked by sequencing the junction, which showed repair patterns typical of NHEJ (Supplementary Figure 4.c). We therefore concluded that the NHEJ system from *B. subtilis* was functional in *S. aureus.*

Supplementary Note 2: Model for NHEJ system detection with MacsyFinder

<system inter_gene_max_space="5" min_mandatory_genes_required="1" min_genes_required="1">
<gene name="ku" presence="mandatory" loner="1"/>
<gene name="ligD1" presence="accessory"/>
<gene name="ligD2" presence="accessory"/>
<gene name="ligD3" presence="accessory"/>
</system>

Supplementary References

1. Horinouchi, S. & Weisblum, B. Nucleotide Sequence and Functional Map of pC194, a Plasmid That Specifies Inducible Chloramphenicol Resistance. *J. Bacteriol.* **150,** 815–825 (1982).
2. Byeon, W. H. & Weisblum, B. Post-transcriptional regulation of chloramphenicol acetyl transferase. *J. Bacteriol.* **158,** 543–550 (1984).
3. Khan, S. a, Adler, G. K. & Novick, R. P. Functional origin of replication of pT181 plasmid DNA is contained within a 168-base-pair segment. *Proc. Natl. Acad. Sci. U. S. A.* **79,** 4580–4584 (1982).
4. Ogasawara, N. Systematic function analysis of Bacillus subtilis genes. *Res. Microbiol.* **151,** 129–134 (2000).
5. de Vos, W. M. Gene cloning in lactic streptococci. *Netherlands Milk Dairy J.* **40,** 141–154 (1986).
6. Husmann, L. K., Scott, J. R., Lindahl, G. & Stenberg, L. Expression of the Arp protein, a member of the M protein family, is not sufficient to inhibit phagocytosis of Streptococcus pyogenes. *Infect. Immun.* **63,** 345–348 (1995).
7. Charpentier, E. *et al.* Novel Cassette-Based Shuttle Vector System for Gram-Positive Bacteria Novel Cassette-Based Shuttle Vector System for Gram-Positive Bacteria. *Appl. Environ. Microbiol.* **70,** 6076–6085 (2004).
8. Heler, R. *et al.* Cas9 specifies functional viral targets during CRISPR–Cas adaptation. *Nature* **519,** 199–202 (2015).
9. Goldberg, G. W., Jiang, W., Bikard, D. & Marraffini, L. a. Conditional tolerance of temperate phages via transcription-dependent CRISPR-Cas targeting. *Nature* **4,** 633–637 (2014).
