# Supplementary material for: Inhibition of NHEJ repair by type II-A CRISPR-Cas systems in bacteria
Source: Nat Commun. 2017 Dec 12;8:2094. doi: 10.1038/s41467-017-02350-1 (PMC5727150; doi:10.1038/s41467-017-02350-1)
Supplement: Supplementary file 3 — Description of Additional Supplementary Files [file 41467_2017_2350_MOESM3_ESM.pdf]

Description of Additional Supplementary Files

File Name: Supplementary Data 1
Description: Number of Type II CRISPR-Cas systems and NHEJ systems in bacterial and archaeal genomes
